# Polymyxin B1 within the *E. coli c*ell envelope: insights from molecular dynamics simulations

**DOI:** 10.1007/s12551-021-00869-8

**Published:** 2021-11-22

**Authors:** Dhanushka Weerakoon, Kamen Petrov, Conrado Pedebos, Syma Khalid

**Affiliations:** 1 School of Chemistry, University of Southampton, Southampton, SO17 1BJ UK; 2 Hertford College, University of Oxford, Oxford, OX1 3BW UK; 3 Department of Biochemistry, University of Oxford, Oxford, OX1 3QU UK

**Keywords:** Polymyxins, Bacterial Membranes, Molecular dynamics

## Abstract

Polymyxins are used as last-resort antibiotics, where other treatments have been ineffectual due to antibiotic resistance. However, resistance to polymyxins has also been now reported, therefore it is instructive to characterise at the molecular level, the mechanisms of action of polymyxins. Here we review insights into these mechanisms from molecular dynamics simulations and discuss the utility of simulations as a complementary technique to  experimental methodologies.

## Introduction

Polymyxins are often used as last-resort antibiotics in cases in which others have failed. In particular they are used to treat infections caused by the Gram-negative ‘superbugs’: *Acinetobacter baumannii*, *Pseudomonas aeruginosa* and *Klebsiella pneumoniae*. (Velkov et al. 2016; Zavascki et al. 2007; Evans et al. 1999) Here we review mechanistic findings of polymyxin B1 (PMB1) and polymyxin E (colistin) permeation across the cell envelope of Gram-negative bacteria from computational studies; we note that this is not an exhaustive review but serves to highlight some key findings from simulation studies.

**Fig. 1:**
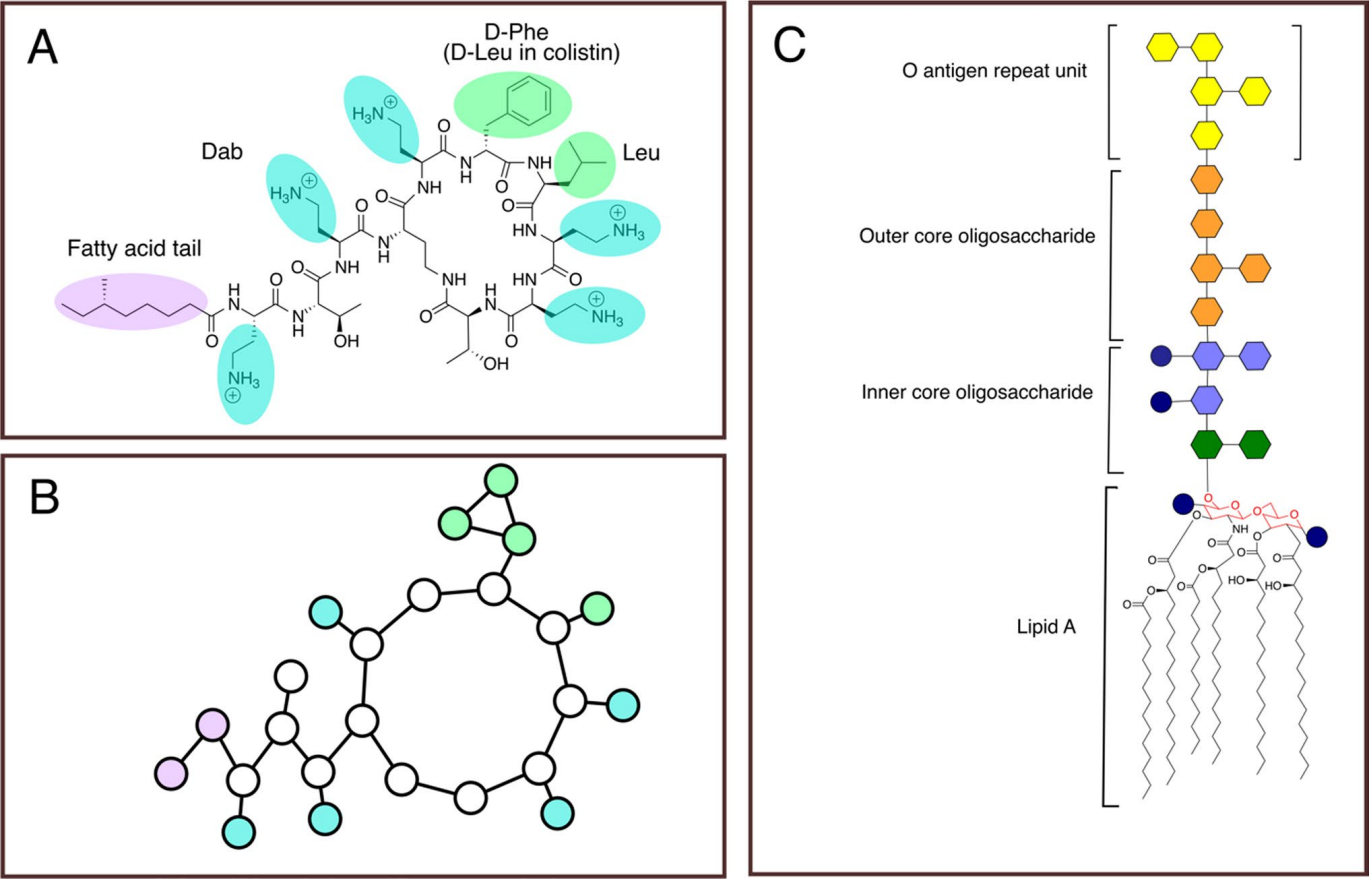
**A** Structure of PMB1 (blue, side chains of Dab residues; light green, side chains of hydrophobic residues; light purple, fatty acid tail). **B** A coarse-grained (Martini2) representation of PMB1 (same colour scheme as Fig. 1A). **C** Structure of *E. coli* LPS (type 1 lipid A, R3 core)(Hsu et al. 2017): chemical structure of type 1 lipid A is given (glucosamine headgroup highlighted in red), sugars are represented by coloured hexagons, and phosphates are represented by navy circles (green, 2-keto-3-deoxyoctulosonate (Kdo); purple, D-manno-heptose; orange, outer-core hexoses (D-glucose, D-galactose, N-acetyl-D-glucosamine); yellow, O-antigen sugars)

Polymyxins are a class of lipopeptide antibiotics produced by *Paenibacillus polymyxa*, as a defence mechanism against Gram-negative bacteria found in soil. (Benedict and Langlykke 1947) The general structure of polymyxins is a heptapeptide loop, a tripeptide side chain and a fatty acid chain (Fig. 1A). Five α,γ-diaminobutyric acid (Dab) residues confer an overall charge of +5 *e*. PMB1 and colistin are the most commonly used polymyxins in the clinic, their structures differ by a single residue (D-Phe/D-Leu respectively)(Velkov et al. 2010; Storm et al. 1977), and they are thought to share similar mechanisms of action. (Kwa et al. 2007) The most widely accepted theory of polymyxin movement across the outer membrane is that this occurs via a ‘self-promoted uptake’(Hancock 1984; Hancock 1997; Zhang et al. 2000; Hancock and Bell 1988) mechanism, while theories of cell lysis by polymyxins include barrel-stave insertion(Hancock 1997; Shai 1999; Dupuy et al. 2018; Khondker and Rheinstädter 2020), carpet insertion(Shai 1999; Khondker and Rheinstädter 2020; Deris et al. 2014) and polymyxin-mediated phospholipid (PL) exchange between the outer and inner membranes. (Clausell et al. 2007; Cajal et al. 1996) Insights into the mode of action of polymyxins have generally come from experimental studies, but in recent years, computational methods such as molecular dynamics (MD) simulations have become increasingly utilised to probe polymyxin behaviour. The recent increase in the number of MD studies of polymyxins has been enabled by the development of detailed atomistic and coarse grained (CG) models of the outer membrane (Lee et al. 2019; Hsu et al. 2017; Hsu et al. 2016). MD simulations provide details at the atomistic or near-atomistic level of resolution (Fig. 1B), albeit with the caveat that the simulated timescales are relatively short (μs).

## Polymyxin interactions with the outer membrane

The outer membrane of Gram-negative bacteria contains lipopolysaccharide (LPS) in the outer leaflet and a mixture of PLs in the inner leaflet. The precise composition of both leaflets varies depending on the bacterial species. (Sohlenkamp and Geiger 2016; Di Lorenzo et al. 2021) LPS is a complex glycolipid consisting of a lipid A moiety (with 4–6 acyl tails) attached to an oligosaccharide core, which may be attached to a highly variable O-antigen polysaccharide (Fig. 1C). LPS molecules are non-covalently cross-linked by divalent cations (Ca^2+^ or Mg^2+^). The sugar regions of LPS present an energetic barrier to hydrophobic molecules, while the lipid A tails present a barrier to polar molecules. (Carpenter et al. 2016)

It is thought that polymyxins move across the outer membrane via the ‘self-promoted uptake’ mechanism, first proposed by Hancock et al. (Hancock 1984; Hancock 1997; Zhang et al. 2000; Hancock and Bell 1988). The first step in the mechanism is binding of the polymyxin cationic moieties to the anionic groups of LPS, with concomitant displacement of divalent cations that cross-link LPS molecules. (Teuber and Bader 1976; Wiese et al. 1998) The sequence of events following this is unclear but is thought to involve destabilisation of the outer membrane structure, thereby easing subsequent uptake of polymyxins. This destabilisation may take the form of transient ‘openings’ in the outer membrane(Hancock 1997; Hancock and Bell 1988) through which other molecules can pass, or it may be linked to increased fluidity of the outer membrane. (Hancock 1997) Experimental studies(Deris et al. 2014; Salazar et al. 2017; Ryder et al. 2014; Halder et al. 2015), particularly recent biophysical investigations of assembled outer membrane models(Dupuy et al. 2018; Han et al. 2018; Paracini et al. 2018; Oh et al. 2017), allow the study of polymyxin-outer membrane interactions, but atomistic level resolution cannot be readily achieved.

Data from molecular simulations supports the initial stages of the proposed ‘self-promoted uptake’ mechanism while providing additional details on subsequent stages. Based on this, the movement of polymyxins through the outer membrane of polymyxin-susceptible bacterial strains may involve the following stages:

1. Interaction with LPS sugar residues(Berglund et al. 2015; Jefferies et al. 2017; Fu et al. 2020; Ongwae et al. 2020; Feigman et al. 2018; Santos et al. 2017; Jiang et al. 2021a)
2. Polymyxin binding to lipid A headgroups(Berglund et al. 2015; Jefferies et al. 2017; Fu et al. 2020; Ongwae et al. 2020; Feigman et al. 2018; Santos et al. 2017; Jiang et al. 2021a; Li et al. 1862; Jiang et al. 2020a; b; Li et al. 2020; Jiang et al. 2021b)
3. Polymyxin insertion (insertion of polymyxin hydrophobic moieties into hydrophobic membrane core)(Berglund et al. 2015; Jefferies et al. 2017; Fu et al. 2020; Ongwae et al. 2020; Li et al. 1862; Jiang et al. 2020a; b; Li et al. 2020; Jiang et al. 2021b)
4. Polymyxin translocation (movement of the full polymyxin molecules into and through the hydrophobic membrane core)(Jefferies et al. 2017; Jiang et al. 2020a; b; Jiang et al. 2021b).

### Interaction of polymyxin with LPS

#### Polymyxins form long-lived, electrostatic interactions with the saccharide moieties

In atomistic (we use all atom and united atom under the umbrella term ‘atomistic’) and coarse-grained simulations, polymyxins have been shown to directly interact with ReLPS (lipid A + Kdo) sugars, forming interactions with Kdo carboxyl groups via their Dab residues(Berglund et al. 2015; Jefferies et al. 2017; Santos et al. 2017) and with inner core heptose phosphates in membranes with more complete LPS models. (Ongwae et al. 2020; Feigman et al. 2018) Atomistic simulations of the interaction between PMB1 and outer membrane models show that PMB1 Dab residues are able to cross-link LPS Kdo sugars, with each PMB1 forming an average of ~8 hydrogen bonds with LPS. (Berglund et al. 2015) PMB1s bind to the LPS sugars for ~1.8 μs, preventing PMB1s from reaching the lipid A headgroups during the 2.2 μs simulation. The longevity of such interactions is also reported from other MD simulations(Jefferies et al. 2017; Ongwae et al. 2020) and indicates that the LPS sugars may act as a kinetic barrier to polymyxins permeating to the LPS headgroups. In 3-5 μs unbiased CG simulations of PMB1s interacting with an asymmetric RaLPS:PL bilayer (PMB1/LPS ratios of 1:4, 1:2, 1:1), PMB1s are mostly found to not penetrate deeper than the heptose sugars of RaLPS. (Jiang et al. 2021a) As part of this study, a one dimensional free energy profile generated for the movement of a single PMB1 from the 1:1 LPS: PL bilayer indicates that while the free energy initially decreases on moving through the LPS sugar region, it rapidly increases on moving into the Kdo sugar region. (Jiang et al. 2021a)

#### Extensive aggregation on LPS saccharides occurs in some simulation studies

Interestingly in some atomistic and coarse-grained simulations of ReLPS-containing systems, PMB1 aggregation on the membrane is observed, (Berglund et al. 2015; Fu et al. 2020) while in other comparable simulations, extensive aggregation is not observed. (Jefferies et al. 2017; Santos et al. 2017) This may be due to the lower polymyxin/LPS ratios (5:100(Jefferies et al. 2017), 6:100 (Santos et al. 2017) used in the latter simulations compared to those used in simulations in which aggregation was observed (38:100(Berglund et al. 2015), (9:100, 12:100, 15:100) (Fu et al. 2020)). These ratios are worked out simply by using the total number of polymyxin and LPS molecules reported for each simulation system. It should be noted that the number of water molecules, and therefore PMB1 concentrations, in each simulation study is not always reported; thus, quantitative comparisons regarding concentrations are not possible. This is further complicated by the issue of conversion between different levels of resolution.

#### Counterions play an important role in determining polymyxin-LPS interactions

Jefferies et al. perform CG simulations of PMB1 interacting with a symmetric ReLPS bilayer neutralised with either Ca^2+^ or Na^+^ ions. (Jefferies et al. 2017) In these simulations, it takes less time for PMB1 to permeate through the ReLPS sugars to reach the Lipid A headgroups when Na^+^ ions are present compared to Ca^2+^. Furthermore, PMB1 is able to penetrate slightly deeper into the outer membrane in the presence of Na^+^.

##### Polymyxin binding to lipid A headgroups

**Fig. 2:**
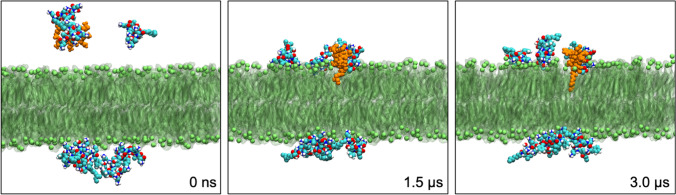
PMB1 (cyan, white, blue, red) interaction with a bilayer containing lipid A (green) in both leaflets. Initially the PMB1 molecules are in the water layers in either side of the bilayer. A single PMB1 molecule (orange) is observed to bind to the lipid A headgroups within 1μs and then insert its acyl tail into the bilayer by 3 μs(Berglund et al. 2015)

Having negotiated the layers of sugars, PMB1 next encounters the lipid A headgroups which are cross-linked by divalent counterions (Mg^2+^, Ca^2+^). In agreement with structure-activity relationship data(Velkov et al. 2010), simulations show that PMB1 binding to lipid A headgroups involves electrostatic interactions between the cationic PMB1 Dab residues and the anionic lipid A phosphate groups (Fig. 2) (Berglund et al. 2015; Jefferies et al. 2017; Fu et al. 2020; Ongwae et al. 2020; Feigman et al. 2018; Santos et al. 2017; Li et al. 1862; Jiang et al. 2020a; Jiang et al. 2020b; Li et al. 2020) Counterion displacement upon PMB1 binding was proposed as a key step of the original ‘self-promoted uptake’ mechanism. (Hancock 1984; Hancock 1997; Zhang et al. 2000; Hancock and Bell 1988) The strongest evidence of this from MD is provided by measurement of Ca^2+^ ion diffusion rates before and after polymyxin binding from atomistic simulations of PMB1 binding to a variety of lipid A/ReLPS bilayers. (Santos et al. 2017) In this study, Ca^2+^ ion lateral diffusion coefficients increase by at least 2-fold following PMB1 binding, with the increase being larger for lipid A bilayers compared to ReLPS bilayers. Fu et al. report similar observations in their CG simulations with the Ca^2+^ dissociation rate increasing with increasing PMB1 concentration. (Fu et al. 2020)

#### Free energy changes upon polymyxin binding

Jefferies et al. report potential-of-mean-force (PMF) free energy profiles of PMB1 moving from bulk solution to a ReLPS bilayer from CG simulations. (Jefferies et al. 2017) The results indicate that as polymyxins move through the ReLPS sugars towards the lipid A phosphate groups, the free energy decreases due to the favourable enthalpic contributions, despite the entropy change being unfavourable. The unfavourable entropy change is attributed to membrane ordering, specifically phosphate crystallisation induced by PMB1 binding, rather than decrease in conformational entropy of PMB1. Interestingly atomistic PMF profiles generated by Jiang et al. show favourable enthalpy change of polymyxin-lipid A binding, −17 to −13 kJ/mol. (Jiang et al. 2020a, b) The differences in simulation resolution, length and protocols makes direct, quantitative comparison of these studies difficult.

### Polymyxin insertion into outer membrane core

#### Difficulties in sampling polymyxin insertion

In both simulations and experiment, polymyxins are found to insert their hydrophobic residues and/or acyl tails into the hydrophobic membrane core after binding to the lipid A headgroups (Fig. 2). (Han et al. 2018; Paracini et al. 2018) In unbiased atomistic and coarse-grained simulations, it is difficult to sample insertion events due to the slow diffusion of LPS. (Berglund et al. 2015; Jefferies et al. 2017; Fu et al. 2020) Replacing Ca^2+^ ions with Na^+^ ions(Berglund et al. 2015; Jefferies et al. 2017), reducing LPS to just lipid A molecules(Berglund et al. 2015; Santos et al. 2017; Li et al. 1862; Jiang et al. 2020a, b; Jiang et al. 2021b; Santos et al. 2017) and using symmetric bilayers (the latter strategy provides two leaflets of LPS; therefore, simulation time is not ‘wasted’ by PMBs interacting with PLs)(Berglund et al. 2015; Jefferies et al. 2017; Fu et al. 2020; Ongwae et al. 2020; Santos et al. 2017; Li et al. 1862) are common strategies used to bypass this difficulty. These strategies have been used to observe (mostly) isolated insertion events in a select few simulations into lipid A bilayers (atomistic)(Berglund et al. 2015; Li et al. 2020), ReLPS (CG) bilayers(Jefferies et al. 2017; Fu et al. 2020) and in one case into a full atomistic LPS bilayer(Ongwae et al. 2020). Polymyxin insertion is also seen in simulations employing enhanced simulations such as umbrella sampling to calculate free energies. (Jefferies et al. 2017; Jiang et al. 2020a, b; Jiang et al. 2021b) PMF profiles from these simulations (both atomistic and CG) predict that polymyxin translocation towards the hydrophobic membrane core is accompanied by an increase in free energy.

#### Folded amphipathic structure may be important for translocation

Interestingly in two studies in which atomistic steered-MD and umbrella sampling are employed,(Jiang et al. 2020a, b) following insertion, polymyxins adopt a folded amphipathic structure during translocation, which enables them to simultaneously maintain interactions with lipid A phosphates (via their Dab residues) and with lipid A acyl tails (via their hydrophobic moieties). This structure is in agreement with a model of PMB1 interacting with lipid A derived from NMR data. (Mares et al. 2009) Furthermore, in a very recent combined in silico and in vitro study, enhanced sampling simulations are used to observe the translocation of a peptide (PMB_3_), which has similar bactericidal activities to the polymyxins but differs structurally by the absence of a chiral methyl group on the acyl tail, with alanine substitutions at various positions. (Jiang et al. 2021b) This study highlights the role of specific residues in translocation and provides a good example of correlation of in silico data with experimental measures of OM disorganisation and MICs (in vitro*)* in *A. baumannii*. (Jiang et al. 2021b)

### Polymyxin permeation towards the inner leaflet of the outer membrane

Following binding and insertion into the core of the outer membrane, the next steps are movement through the inner leaflet to enter the periplasm. (Hancock and Bell 1988) Jiang et al. report the molecular details of membrane disruption on polymyxin translocation. Their simulations show that polymyxin translocation is accompanied by 3-4 bound lipid A molecules being pulled into the membrane core. (Jiang et al. 2020a, b) This creates a pore-like structure in the membrane through which water and ions can permeate. Subsequently equilibrium simulations were initiated from a snapshot of one of the umbrella sampling windows in which the pore was present. Additional colistin molecules were added to the water on the lipid A side of the membrane. The pore persisted throughout the simulation and one colistin molecule spontaneously entered it from solution. (Jiang et al. 2020a)

Notably, when colistin is pulled past the centre of the membrane, towards the PLs of the inner leaflet, the pore closes in the final windows of umbrella sampling simulations, with lipid A molecules returning to the outer leaflet. (Jiang et al. 2020b) To our knowledge, this is to date the only example of late-stage ‘self-promoted uptake’ observed in an MD simulation, in which the membrane damage induced by polymyxin aids the uptake of another polymyxin. Biophysical data on the interaction of PMB1 with an asymmetric PL-LPS bilayer shows that partial polymyxin translocation occurs, with perturbation of membrane structural properties (reduction of membrane thickness, mixing of lipids between leaflets). (Paracini et al. 2018) Other experimental data shows conclusively that the outer membrane is not lethally damaged by polymyxin permeation; thus, the persistence of pores in simulations is likely a result of short simulation timescales and the slow-moving nature of LPS. (Sabnis et al. 2021; MacNair et al. 2018)

We note here that a number of simulation studies exist in which the interaction of polymyxins with modified LPS-containing membranes are reported: these are beyond the scope of this short review. (Salazar et al. 2017; Santos et al. 2017; Li et al. 1862; Jiang et al. 2020b; Li et al. 2020)

### Dynamics of polymyxins in the periplasm

**Fig. 3:**
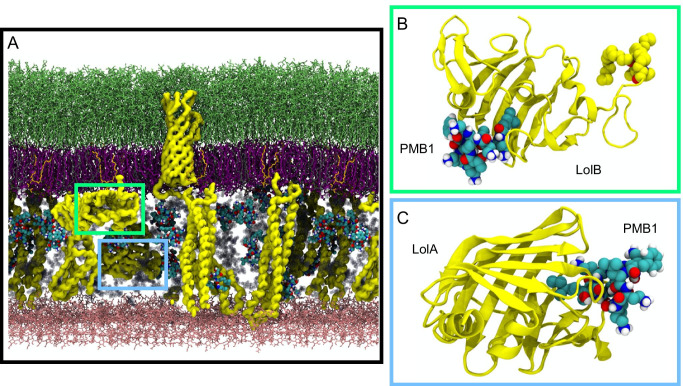
**A** Crowded model of a portion of the cell envelope, showing the many elements that compose this compartment. PMB1 is shown in cyan, blue, red and white spheres; outer membrane is shown in sticks with LPS in green, 1-palmitoyl 2-cis-vaccenyl-phosphatidylethanolamine (PVPE) in purple, 1-palmitoyl 2-cis-vaccenic phosphatidylglycerol (PVPG) in orange, 1-palmitoyl 2-cis-vaccenic 3-palmitoyl 4-cis-vaccenic diphosphatidylglycerol (PVPV) in grey as well as the cell wall (in pink); proteins are shown in yellow surface; osmolytes are depicted as silver spheres, and part of them have been omitted to aid visualisation. **B** and **C** show the main binding modes of PMB1 to LolB and LolA, respectively. In both cases, PMB1 uses its hydrophobic tail to insert in the lipoprotein carriers binding site during the 500-ns simulations performed

Having permeated across the outer membrane, polymyxins next encounter the aqueous periplasm. This cell envelope compartment is densely crowded with proteins and osmolytes and also contains the cell wall (peptidoglycan). To our knowledge at the time of writing this review, there is only one published simulation study of polymyxins in the periplasm. Pedebos et al report atomistic simulations of PMB1 in a range of periplasm models crowded to differing extents, from very dilute to realistic crowded volume fractions (Fig. 3A). (Pedebos et al. 2021) Long-lived associations between PMB1 and various periplasmic proteins and the cell wall are reported. From a mechanistic perspective, the most interesting finding is the spontaneous binding of PMB1 (via its acyl tail) to the lipoprotein carriers LolA (Fig. 3C) and LolB (Fig. 3B). It is hypothesised that LolA carrying polymyxins across the periplasm may be one mechanism via which these antibiotics reach the inner membrane. While currently there is no direct evidence to either support or disprove this simulation-generated hypothesis, it should be noted that transcriptomics data for *E. coli* does show increased LolA transcription on PMB1 treatment, providing some indirect support. (Tao et al. 2012)

### Dynamics of polymyxins in the inner membrane

Cell lysis occurs as a result of polymyxin action on the inner membrane. Some insights into this action have been gleaned from MD simulations. In atomistic simulations of PMB1 molecules interacting with a symmetric bilayer composed of a mixture of PLs, Berglund et al. find polymyxins initially from hydrogen bonds with PLs via their Dab residues before spontaneously inserting their acyl tails into the membrane core within 100 ns of binding to the membrane (Fig. 2). (Berglund et al. 2015) This is followed by translocation towards the inner leaflet, in which polymyxins adopt the folded amphipathic conformation seen in other experimental(Mares et al. 2009) and simulation studies(Jiang et al. 2020a, c), accompanied by extensive membrane disruption. (Berglund et al. 2015) No aggregation is reported in these simulations, with polymyxins translocating as monomers, bearing similarities to the barrel-stave model of peptide insertion. (Shai 1999; Khondker and Rheinstädter 2020) In coarse-grained simulations of PMB1 interacting with a compositionally simpler inner membrane model at varying polymyxin/PL ratios, Fu et al. observe similar polymyxin binding and insertion behaviour with the degree of insertion depending on polymyxin/PL ratio. (Fu et al. 2020) However, here polymyxin translocation and membrane disruption are not observed. Instead polymyxins aggregated on the membrane, and an increase in bilayer bending rigidity is reported(Fu et al. 2020). The observed increase in area per lipid and reduction in membrane thickness with increasing polymyxin/PL provides some support for the carpet model of peptide insertion. (Shai 1999; Khondker and Rheinstädter 2020) Thus, the two simulation studies show similarities but also some striking differences.

To determine the membrane physical properties affecting PMB1-PL membrane interactions, Khondker et al. perform atomistic simulations of mixed PL bilayers with varying ratios of the constituent lipids and polymyxin/PL ratios (2:100–10:100). Some simulations are initiated from configurations from X-ray data. (Khondker et al. 2019) While the lipids used are not representative of those found in Gram-negative bacteria, they are chosen to assess the impact of membrane surface charge (POPS (1-palmitoyl-2-oleoyl-sn-glycero-3-phospho-L-serine)) and lipid acyl chain packing (DMPS (1,2-dimyristoyl-sn-glycero-3-phospho-L-serine)) on PMB1-PL interaction. (Khondker et al. 2019) Polymyxins are found to insert into membranes with POPS, with the extent of insertion increasing with POPS fraction and hence increasing surface charge, in a mechanism similar to that observed by Berglund et al. (Berglund et al. 2015) However, with DMPS-containing bilayers, polymyxins are found to lie flat on the surface of the membrane, with aggregation occurring more readily on DMPS-containing bilayers than POPS-containing bilayers. (Khondker et al. 2019) This mechanism of interaction has some similarities to that observed by Fu et al. (Fu et al. 2020) The authors use their data to define a mathematical model for predicting polymyxin insertion depth. (Khondker et al. 2019)

We note that *A. baumannii* can form an outer membrane without LPS, which is believed to confer polymyxin resistance to the bacterium. (Moffatt et al. 2010) The LPS-free outer membrane is effectively a PL bilayer, and hence simulation studies of its interactions with colistin merit discussion. In umbrella sampling simulations, Jiang et al. show that colistin binding to their LPS-deficient model outer membrane model is energetically unfavourable, due to the lower surface charge relative to LPS-containing bilayers(Jiang et al. 2020b) This is in broad agreement with recent work by Edwards and co-workers which has shown that polymyxins pass through the inner membrane by interacting with the small quantities of LPS present in it. (Sabnis et al. 2021; Humphrey et al. 2021) Zhu et al. also perform atomistic simulations of symmetric bilayers representing LPS-deficient *A. baumannii* membranes with varying proportions of PG lipids (lipids with phosphatidylglycerol headgroup). At PG concentrations >35:65 (PG: other lipids) colistin molecules are found to bind preferentially to the anionic PG lipids, leading to a reduction in lipid lateral diffusion. (Zhu et al. 2020) No insertion or translocation is reported, but this is likely due to the short timescales (100 ns) of the simulations. (Zhu et al. 2020) Both the Jiang and Zhu studies highlight the importance of PL charge in polymyxin-PL interactions, in agreement with the work of Khondker et al. (Khondker et al. 2019)

Fu et al. report CG simulations of the interaction of a vesicle and planar bilayer (both composed of a mixture of 1-palmitoyl-2-oleoyl-sn-glycero-3-phosphoethanolamine (POPG) and 1-palmitoyl-2-oleoyl-sn-glycero-3-phosphoethanolamine (POPE)) in the presence/absence of a single PMB1. In both scenarios, the vesicle and bilayer contact and exchange lipids, but contact occurs faster in the presence of PMB1 (~18 ns vs ~260 ns), across 3 repeat simulations. PMB1 acts as a contact site between the vesicle and the planar bilayer by adopting a conformation in which the hydrophobic moieties of the antibiotic insert into the vesicle and bilayer. (Fu et al. 2020) This provides support to the theory that polymyxin-mediated PL exchange between inner and outer Gram-negative membranes may contribute to cell lysis. This theory originated from experimental work including studies which showed that PMB1, but not polymyxin B nonapeptide (PMB1 without the fatty acyl tail) is able to induce fast PL exchange in POPG(Clausell et al. 2007; Cajal et al. 1996; Clausell et al. 2006) and POPE/POPG(Grau-Campistany et al. 2016) vesicles. Interestingly, recent transcriptomics data shows upregulation of genes involved in the Mla system on polymyxin treatment, which is involved in maintaining PL levels in both membranes(Henry et al. 2015), providing some support for polymyxin-mediated PL exchange. (Mann et al. 2021)

### Summary

In summary, MD simulations have provided mechanistic insights into the action of polymyxins on both the inner and outer membranes of Gram-negative bacteria. There has been less work reported on the dynamics of polymyxins in the periplasm, but early work suggests a complex series of interactions with native proteins and the cell wall. Thus, details of the dynamic behaviour of polymyxins within all three compartments of the cell envelopes of Gram-negative behaviour are now beginning to emerge. Nevertheless, we still do not have a comprehensively characterised molecular-level picture of the complete process of cell lysis as some questions remain about the individual steps involved: (i) how do polymyxin aggregates cross the outer membrane, given the outer membrane is not completely disrupted by polymyxin permeation? (ii) What is the precise mechanism of their action at the inner membrane? (Does it involve any interactions with native membrane proteins?) (iii) How do polymyxins cross the periplasm—as monomers, aggregates, via simple diffusion, via binding to lipoprotein carriers or a combination of all of these?

As we have shown, MD simulations are playing an increasingly significant role in addressing these questions. However, as with all scientific measurement/prediction methods, MD simulations have their own set of limitations and caveats. The most impactful of these for mechanistic studies of biological systems is the relatively short time and length scales that are accessible. This is partly mitigated by CG models, in which the lower resolution is compensated by longer timescales and larger simulation systems. (David et al. 2019) While the CG simulation studies of polymyxins reported in the literature to date have employed the Martini set of CG force fields, encouragingly a number of other CG force fields now exist which are also well suited for the simulation of biological membrane systems. (Orsi and Essex 2011; Machado et al. 2019) The MARTINI force field has the advantages of having a wide library of parameterised lipids including LPS already available and allowing automated system setup for flat membrane, vesicles, nanodiscs and micelles via the CharmmGui webserver. (Hsu et al. 2017) The latest version of the force field, MARTINI3, has an expanded repertoire of particle types, but some bacterial lipids remain to be parametrised. (Souza et al. 2021) One slight caveat with the MARTINI force fields is that the secondary structure of proteins is fixed, so conformational changes in proteins are not accessible (other than inter-domain motions). In this context, this is only a slight caveat as such conformational changes are likely not of particular importance to the mechanistic studies of polymyxins. However, this issue is addressed by the SIRAH(Machado et al. 2019) force field in which secondary structure of proteins is not fixed.

Ultimately, it is desirable to go beyond a mechanistic understanding of polymyxins such that computational methods can be employed to inform the design of novel, more potent antibiotics, particularly as resistance to polymyxins has now emerged. (Binsker et al. 2021; Jeannot et al. 2017) To achieve this, calculations that provide quantitative comparisons of, e.g. free energies of membrane permeation of de novo designed candidate antibiotics are required. Currently, such comparisons are particularly problematic for studies of the outer membrane due to the slow-moving nature of LPS. (Shearer et al. 2020) Thus, methodological advancements are needed to enable more efficient convergence of free energy calculations using, for example, metadynamics and umbrella sampling such that high-throughput comparative computational studies can be performed. (Armacost et al. 2020) The intense research efforts into improved computational methodologies combined with ever-increasing availability of high-performance computing resources are highly likely to soon deliver the capabilities for simulation-informed de novo antibiotic design.
